# Therapeutic effects of Sheng Xue Fang in a cyclophosphamide-induced anaemia mouse model

**DOI:** 10.1080/13880209.2021.1941133

**Published:** 2021-06-27

**Authors:** Lu Dou, Xue Gong, Qing Wu, Fangzheng Mou

**Affiliations:** a Central Laboratory, Chongqing University Three Gorges Hospital, Chongqing, People's Republic of China; b Internal Medicine of Traditional Chinese Medicine, Chongqing University Three Gorges Hospital, Chongqing, People's Republic of China

**Keywords:** Network pharmacology, red blood cell, haematopoiesis

## Abstract

**Context:**

Sheng Xue Fang (SXF) has been used to treat anaemia for decades with good efficacy.

**Objective:**

To study the effect and possible mechanism of SXF to restore haematopoietic function.

**Materials and methods:**

Balb/c mice (10 per/group, half male, half female) were treated with SXF (three dose groups, 8.5, 17, and 22.1 g/kg) by gavage for 14 days, and cyclophosphamide (80 mg/kg) was injected on days 10–12. Only injection of cyclophosphamide (negative control) or physiological saline (blank control) were included as controls. The spleen and femur were processed for histopathology. Active components and the target of SXF were screened. The target was used for gene enrichment and protein-protein interaction (PPI) analysis.

**Results:**

Red blood cell relative changes in the SXF group (low: −5.50 ± 1.58%; medium: −11.11 ± 4.15%; high: −8.81 ± 2.67%) and relative negative control (26.21 ± 2.51%) significantly increased (all *p* < 0.01) in female mice. Haemoglobin and red blood cell-specific volume showed the same trend. However, SXF did not have significant effects on male mice. Splenic index in the medium group (4.44 ± 0.46%) relative negative control (3.38 ± 0.10%) significantly improved (*p* < 0.01) in female mice. Using network pharmacology, 77 active components and 337 targets were screened from SXF. These targets are closely related to the mitogen-activated protein kinase pathway.

**Conclusions:**

SXF has good clinical application potential. However, the mechanism requires in-depth research. Our findings are of great significance in anaemia treatment and provide a new perspective for Chinese medicine research.

## Introduction

Anaemia is classified as a ‘defined number of red blood cells (RBCs), often accompanied by diminished hemoglobin concentrations and altered RBC morphology’ (Schumann and Solomons 2017). As one of the most common clinical symptoms, it can reduce the quality of life in patients due to fatigue, chest tightness or pain, shortness of breath, and increased heart rate (Liu et al. 2013; Jin et al. 2019; Zhu et al. 2019). Cyclophosphamide (CTX), a commonly used cancer chemotherapy drug, depletes the bone marrow of haematopoietic stem cells (HSCs), resulting in peripheral circulating hemocytopenia (Xu et al. 2014) and other regenerative anaemia symptoms. However, there is no specific medication to improve the blood status in patients with anaemia caused by chemotherapy drugs, and it is necessary to identify a drug that can restore many of these HSCs (Zhu et al. 2019).

Sheng Xue Fang (SXF) is a non-decoction granular formula derived from the family recipe of Professor Bangben Zheng, a traditional Chinese medicine (TCM) practitioner. The components of SXF were selected based on the Qi and blood theory of Chinese medicine, and contain 12 natural medicines, such as *Hedysarum multijugum* Maxim (Fabaceae) (root), *Angelica sinensis* (Oliv.) Diels (Apiaceae) (root), *Polygonatum sibiricum* Delar. ex Redoute (Convallariaceae) (root), *Fructus ligustri* Lucidi (fruit), *Ziziphus jujuba* Mill (Rhamnaceae) (fruit), *Acanthopanax senticosus* (Rupr. et Maxim.) Harms (Araliaceae) (root and stem), *Colla corii* Asini (donkey skin), *Spatholobus suberectus* Dunn (Leguminosae) (stem), *Codonopsis pilosula* (Franch.) Nannf (Campanulaceae) (root), *Atractylodes macrocephala* Koidz (Asteraceae) (stem), *Poria cocos* (Schw.) Wolf (Sclerotium) (Polyporaceae), and *Citrus sinensis* (L.) Osbeck (Rutaceae) (peel). SXF has the effects of invigorating Qi (a TCM theory) and activating blood circulation, and has been used clinically for decades with good efficacy (Da-Rong et al. 2019). However, the functional mechanism of SXF in the treatment of anaemia is unclear.

Network pharmacology considers drug-disease interactions as processes involving multi-components, multi-targets, and multi-pathways involving the interactions of multiple functional molecules and suggests that multi-component drugs that target multiple organs involved in disease work synergistically to achieve better therapeutic results. This is consistent with the dynamic, dialectical, and holistic view of TCM grounded theory (Hopkins 2008; Lei et al. 2018). Given the multi-component and multi-target characteristics of TCM, this study provides credible evidence of the efficacy of SXF treatment in anaemia by exploring the mechanism of SXF using both animal experiments and network pharmacology. SXF was administered to mice by gavage, routine blood tests, and histopathological examination of the spleen and bone marrow were carried out, followed by determination of the active components of SXF and their targets of action. Text mining tools were used to search for genes related to keywords, such as anaemia, and the two were connected to obtain potential target genes for SXF treatment of anaemia. Gene ontology (GO) enrichment analysis and Kyoto encyclopaedia of genes and genomes (KEGG) enrichment analysis, as well as protein-protein interaction (PPI) analysis of target genes, were performed to derive the possible mechanism of action of SXF.

## Materials and methods

### Experimental animals and handling

**Figure 1.**
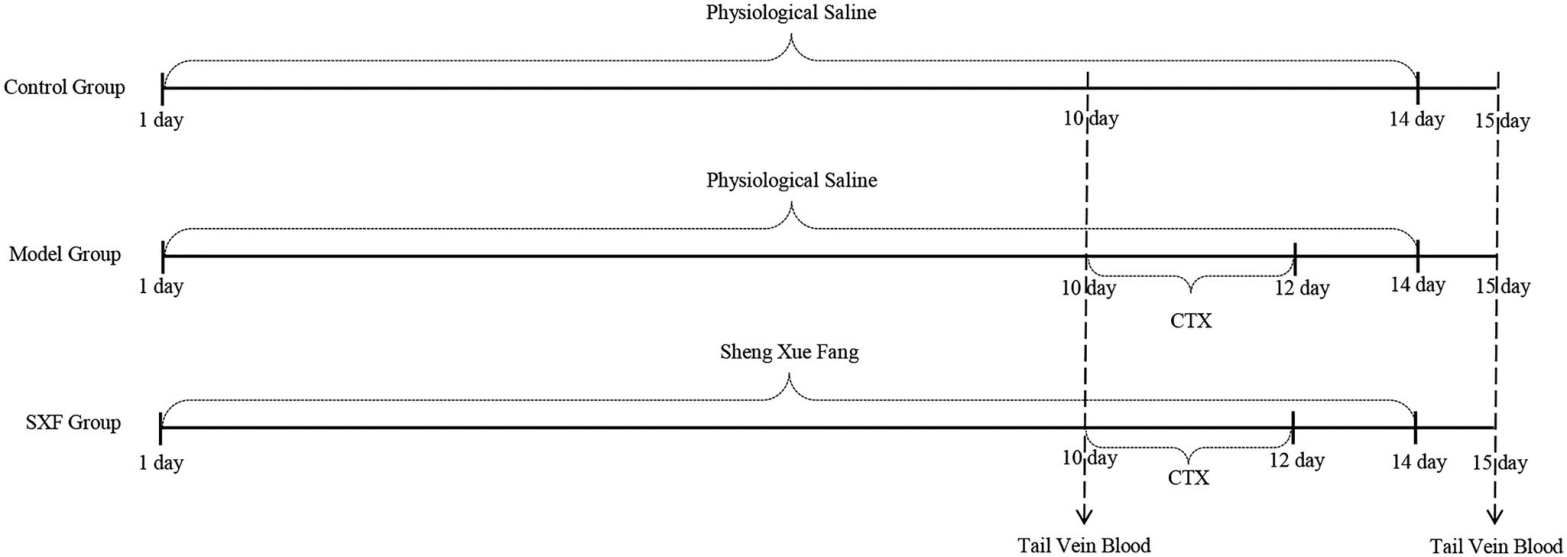
Experimental design.

Specific pathogen-free Balb/c mice, *n* = 50 (10 per/group, half male, half female), weight 20 ± 2 g, and age 6–8 weeks were purchased from Chongqing Medical University (Chongqing, China). The mice were randomly divided into five groups, and after a 7 day acclimatization period, the mice in the control group received saline by gavage once a day for 14 days. Mice in the model group (CTX group) also received the same saline regimen, but on days 10–12, CTX 80 mg/kg was administered intraperitoneally once daily (Zhu et al. 2018). The remaining three groups received SXF by gavage daily for 14 days. SXF was produced by the Pharmacy of National Medical College, Chongqing University Three Gorges Hospital. The doses of SXF in these three groups were 8.5 g/kg (low dose group), 17 g/kg (middle dose group), and 22.1 g/kg (high dose group), respectively. The SXF dose in mice was converted using an equivalent dose ratio table between humans and animals based on body surface area with slight adjustments due to the results of previous experiments (unpublished data). CTX was injected once daily in the three groups at the dose of 80 mg/kg/day on days 10–12. The mice were weighed daily and the drug was administered according to their body weight. Animal experiments were authorized by the Animal Welfare and Ethical Committee of Chongqing University Three Gorges Hospital. The experimental design is shown in Figure 1.

### Routine blood analysis

Blood was collected from the tail vein on days 10 and 15 of the trial for routine analysis, including white blood cells (WBCs), RBCs, haemoglobin (HGB), and red blood cell-specific volume (HCT) levels. Based on the results of the two measurements, the relative change in routine blood parameters was determined according to (*D*_15_ − *D*_10_)/*D*_10_ × 100%, where *D*_15_ was the measured value on day 15, and *D*_10_ was the measured value on day 10. The smaller the value, the lower the degree of variation in the results.

### Histopathological analysis

Twenty-four hours after the last gavage, the mice were weighed and then sacrificed by dislocation of cervical vertebrae. The splenic index was then calculated. The femur and spleen were fixed with 4% paraformaldehyde solution, embedded in paraffin wax, cut into sections, stained according to the hematoxylin-eosin (HE) staining kit instructions (Beyotime, Beijing, China), and microscopically examined using an inverted fluorescence microscope.

### Screening of SXF active components and action target

Traditional Chinese Medicine Systems Pharmacology Database and Analysis Platform (TCMSP) (https://tcmspw.com/tcmsp.php), a technical platform for the pharmacology of TCM systems, was used to obtain the major chemical constituents of the 12 natural medicines in SXF (Ru et al. 2014). The screening thresholds were set as oral bioavailability (OB) ≥30% and drug-like (DL) ≥0.18 in combination with the five drug-like principles. The drugs that could not be retrieved using TCMSP were searched for using Bioinformatics Analysis Tool for Molecular mechANism of Traditional Chinese Medicine (BATMAN-TCM) (http://bionet.ncpsb.org/batman-tcm/) (Liu et al. 2016). The screened active components were identified using PubChem (https://pubchem.ncbi.nlm.nih.gov/) or Discovery Studio 2020 Client software to determine the chemical structural formula. The target of the active component was then obtained using TCMSP or BATMAN-TCM, where the conditions for BATMAN-TCM target screening were a score cut-off ≥30%, and the adjusted *p*-value was set to 0.01 (*p*-value after Benjamini–Hochberg multiple testing correction).

### Prediction of the target of SXF for anaemia

The UniProt (http://www.uniprot.org/) database was used for the conversion of obtained targets to standard gene names (used ‘*Homo sapiens*’ as the organism). The keywords used were ‘anemia’, ‘erythrocytopenia’, and ‘hemocytopenia’, and online platforms, such as GeneCard (https://www.genecards.org/) (Stelzer et al. 2016), GenCLiP 3 (http://ci.smu.edu.cn/genclip3/analysis.php) (Wang et al. 2019), and DisGeNET (https://www.disgenet.org/) (Pinero et al. 2020), were used to perform text-based searches of genes associated with hemocytopenia. Finally, both the resulting active component targets and the hemocytopenia-related genes from the text search were submitted to the http://bioinformatics.psb.ugent.be/webtools/(Venn/) platform to obtain the two intersection genes. The ‘Natural medicines-Active component’ and ‘Active component-Target’ results were entered into Cytoscape (v3.7.2) software (Shannon et al. 2003) and the ‘Merge’ function was used to construct the ‘Natural medicines–Active component–Target’ network.

### Analysis of biological functions and pathways

The previously obtained gene symbols of the action targets were submitted to Metascape (https://metascape.org/gp/index.html#/main/step1) (Zhou et al. 2019) for the GO enrichment analysis and KEGG enrichment analysis. The chosen enrichment processes were GO biological processes (BP), GO cellular components (CC), GO molecular functions (MF), and KEGG pathway analysis with parameter settings to ‘Min Overlap’ = 3, ‘*p*-Value Cutoff’ = 0.1, and ‘Min Enrichment’ = 1.5. The enrichment results were sorted by count, and the top 10 GO enrichment and the top 20 KEGG enrichment processes were subjected to follow-up analysis. The data were preprocessed and submitted to the online website http://www.bioinformatics.com for mapping.

### Construction and analysis of PPI networks

The obtained protein targets were submitted to String (https://string-db.org/) with the species selected as ‘*Homo sapiens*’. The acquired PPI file was saved in ‘TSV’ format and imported into Cytoscape (v3.7.2) software. After removing isolated targets, PPIs were obtained, and the CytoHubba plug-in was used for key nodes analysis.

### Statistical analysis

All results were expressed as the mean ± standard error. One-way analysis of variance was performed, and if the difference between the groups was significant, then the least significant difference (LSD) method was used to perform multiple comparisons. Statistical Product and Service Solutions software (v25.0) (come from International Business Machines Corporation, New York, the United States) was used for analysis, and GraphPad (v8.01) software (come from GraphPad Software Corporation, California, the United States) was used for drawing.

## Results

### Routine blood analysis

**Figure 2.**
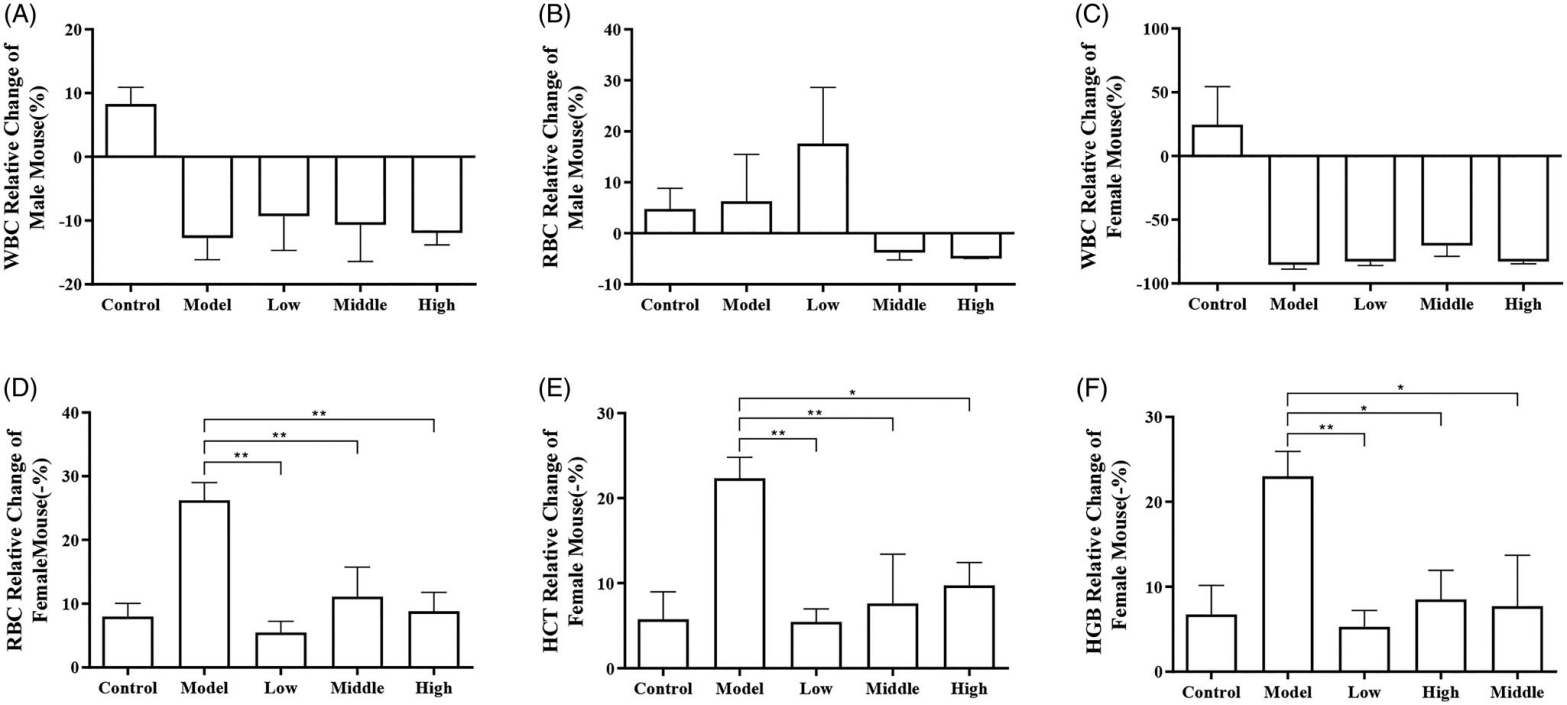
Routine blood parameters. (A) WBC relative change in male mice; (B) RBC relative change in male mice; (C) WBC relative change in female mice; (D) RBC relative change in female mice; (E) HCT relative change in female mice; (F) HGB relative change in female mice. Control: control group; Model: model group (CTX group); Low: low dose group; Middle: middle dose group; High: high dose group. Note: The Y-axis of (D–F) is displayed in −%. *p < 0.05; **p < 0.01.

**Figure 3.**
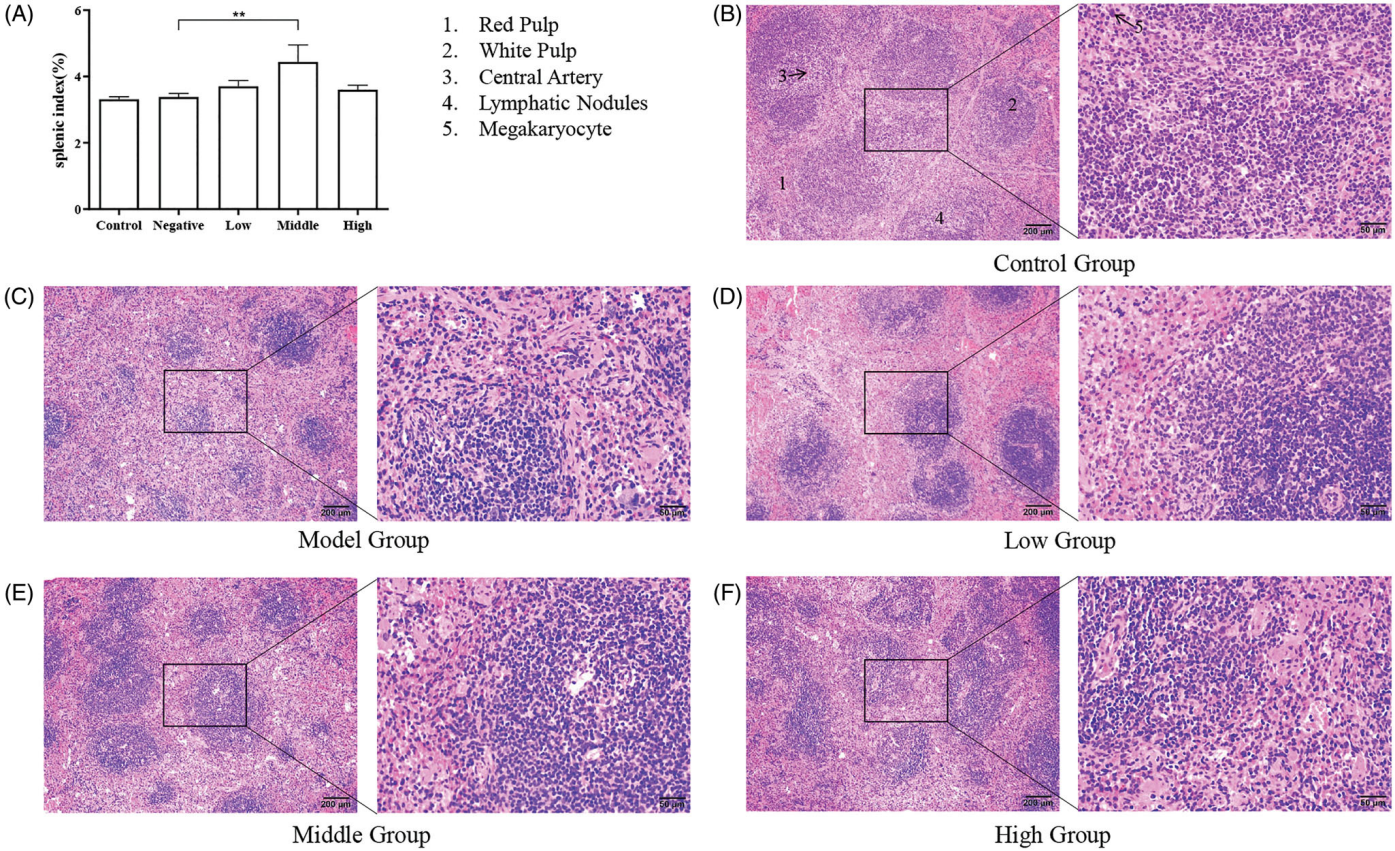
Histopathology of the spleen. (A) Splenic index; (B) Spleen of the control group; (C) Spleen of the model group; (D) Spleen of the low-dose group; (E) Spleen of the middle dose group; (F) Spleen of the high-dose group. *p < 0.05; **p < 0.01.

Following CTX treatment, there was an obvious decrease in RBC and WBC levels in the model group and blank control group (Figures 2(A–F)). After SXF administration, the recovery of WBCs in female mice and male mice did not significantly change (Figures 2(A), 3(C), *p* > 0.05), and RBC recovery in male mice did not significantly change (Figure 2(B), *p* > 0.05). However, the RBC relative change in the SXF groups was low: −5.50 ± 1.58%; medium: −11.11 ± 4.15%; high: −8.81 ± 2.67% and in the negative control was 26.21 ± 2.51% (*p* < 0.05) in female mice (Figure 2(D)). HGB and HCT relative change compared with the model group showed a significant recovery effect (Figures 2(D–F), SXF group all *p* < 0.05).

### Histopathological examination

**Figure 4.**
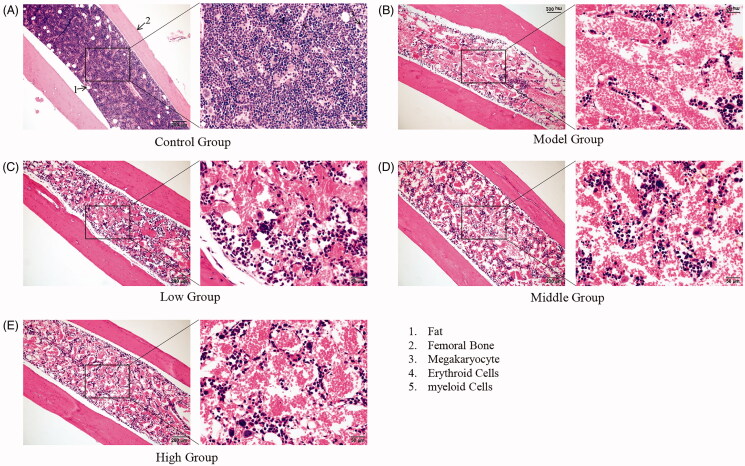
Histopathological observations of femoral bone marrow. (A) Femoral bone marrow of the control group; (B) Femoral bone marrow of the model group; (C) Femoral bone marrow of the low-dose group; (D) Femoral bone marrow of the middle dose group; (E) Femoral bone marrow of the high-dose group.

Sections of female mouse spleen and bone marrow tissue were prepared. Compared with the splenic index of the model group (3.38 ± 0.10%), the index in the SXF groups increased slightly and the middle dose group showed a significant increase (4.44 ± 0.46%, *p* < 0.01); however, the index in the low (3.70 ± 0.16%, *p* > 0.05) and high dose group (3.60 ± 0.12%, *p* > 0.05) was not statistically significant (Figure 3(A)). HE results showed splenic red pulp after CTX administration (Figures 3(B–F)); however, this situation improved to some extent in the SXF groups. The analysis of bone marrow sections showed that after CTX treatment, the number of myeloid and erythroid cells was reduced and the cell density was reduced in each group; however, all SXF-treated groups showed some degree of symptom recovery compared to the model group (Figures 4(A–E)).

### Composition screening and target identification

**Figure 5.**
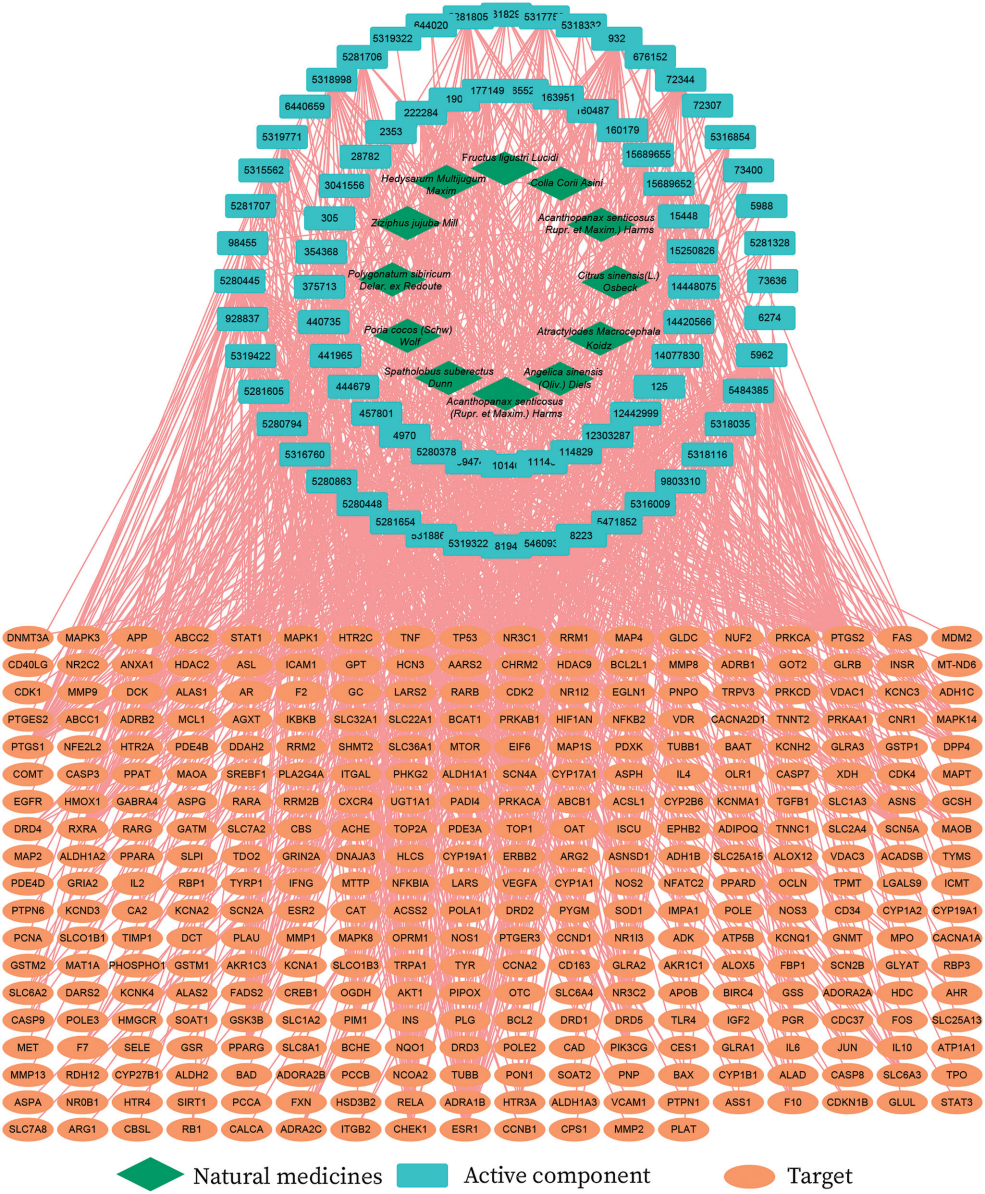
‘Natural medicines–active component–target’ network.

Based on TCMSP and BATMAN-TCM, a total of 77 compounds were obtained based on the screening conditions (Table S1). The 572 protein targets of 77 compounds were also obtained based on TCMSP and BATMAN-TCM, using Uniprot to convert into gene targets. GeneCard, CooLGeN, and DisGeNET obtained a total of 6696 genes, which were then intersected with the gene targets obtained from Uniprot. There were 337 possible human gene targets associated with SXF for elevated WBCs (Table S2 and Figure S1). The above results were used to construct the ‘Natural medicines-Active component-Target’ network (Figure 5). The mean value of the degree of active component nodes was 14.99, and the mean value of the degree of target genes was 3.18. The top 10 components and targets as key nodes are listed in Table 1.

**Table 1.** Top 10 key nodes of natural medicines and actin target.

Pubchem ID/active component	Degree	Target name	Degree
5962/lysine	70	PTGS2	46
5280445/luteolin	56	PTGS1	39
5280863/kaempferol	49	PRKACA	31
932/naringenin	37	AR	29
15689652/7-*O*-methylisomucronulatol	33	ESR1	28
5280378/formononetin	32	ADRB2	24
354368/7-methoxy-2-methyl isoflavone	31	RXRA	24
72344/nobiletin	30	NCOA2	23
5281654/isorhamnetin	30	SCN5A	21
5318998/licochalcone a	29	NOS2	20

**Table 2.** Top 15 in the SXF PPI network ranked by degree.

Rank	Name	Degree	Rank	Name	Degree	Rank	Name	Degree
1	INS	156	2	AKT1	150	3	TP53	134
4	IL6	130	5	MAPK3	125	6	VEGFA	122
7	TNF	115	8	MAPK1	111	9	CASP3	109
10	JUN	106	11	EGFR	105	12	CAT	101
13	FOS	100	14	STAT3	99	15	MAPK8	97

### Enrichment analysis

**Figure 6.**
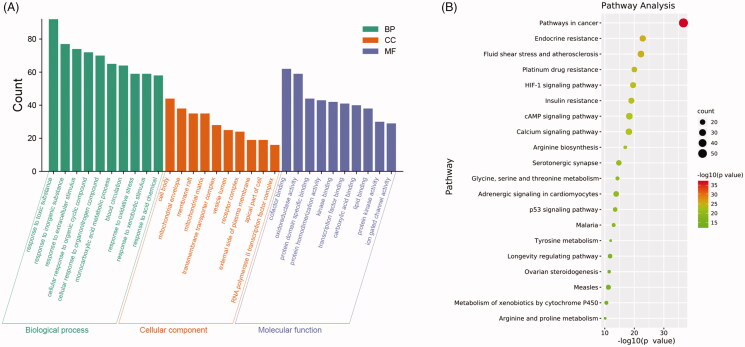
Enrichment analysis. (A) GO enrichment analysis; (B) KEGG enrichment analysis.

We performed a GO analysis of the SXF genes targeted (Table S2 and Figure 6(A)) and found that these genes were predominantly involved in responses to many substances, oxidative stress responses, blood circulation, and some stimulus signals in the BP. In the CC, these genes were mainly involved in cell bodies, mitochondria, vesicles, plasma membranes, and nuclei; in the MF, the genes mainly involved protein (enzyme) binding, transcription factor binding, oxidoreductase activity, and ion gating channel activity. KEGG enrichment results indicated that SXF target genes were mainly enriched in pathways in cancer, the cyclic adenosine monophosphate signalling pathway, calcium signalling pathway, endocrine resistance, p53 signalling pathway, glycine, serine and threonine metabolism, and the HIF-1 signalling pathway (Figure 6(B)).

### PPI analysis

**Figure 7.**
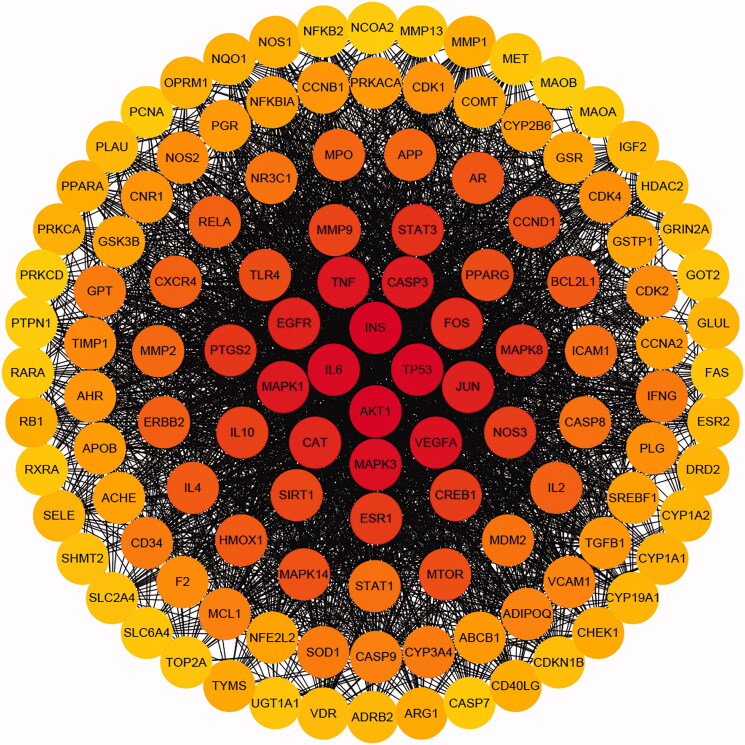
PPI network. The darker the colour, the greater the degree.

Protein targets were submitted to String, yielding a total of 337 nodes and 2742 edges (Figure 7), with an average node degree of 30. Seventy-seven and 122 nodes were greater than the mean. There were four nodal degrees >130, such as insulin (INS) (156), RAC α serine/threonine-protein kinase (AKT1) (150), cellular tumour antigen p53 (TP53) (134), and interleukin-6 (IL6) (130) (Table 2).

## Discussion

The TCM theory proposes that anaemia belongs to categories, such as ‘blood deficiency’, ‘chlorosis’, ‘blood syndrome’, and ‘consumptive disease’ which manifest in dizziness, tinnitus, insomnia, pallor, weakness, and other symptoms. Chinese Materia Medica is derived from natural plants and has high safety, few side effects, and significant efficacy (Wang et al. 2019; Cui et al. 2020; Kahn et al. 2020). SXF has been used in the clinical treatment of anaemia for many years and has been effective in significantly improving symptoms of anaemia (Da-Rong et al. 2019). However, its main active component and target of action are unclear; thus, we conducted the current study. SXF significantly improved RBC and HGB levels, and HCT in female Balb/c mice. However, the improvement was not observed in male mice, and no improvement was observed in WBCs in female mice. No significant WBC-enhancing effect of SXF was observed in these mice, which may be due to species differences between humans and mice. There were also differences between male and female mice regarding RBC increases, suggesting that male and female mice may also show differences in immune resistance and haematopoietic function (Borkar et al. 2020; Merikangas and Almasy 2020; Rubin et al. 2020).

The bone marrow of adult animals has not only haematopoietic functions but also immune defense functions. The bone marrow includes haematopoietic cells at different stages of maturation (Liu et al. 2014; Tedesco et al. 2020). CTX, a chemotherapeutic agent, is a commonly used induction agent in animal models of anaemia, that inhibits bone marrow haematopoiesis to induce chronic aplastic anaemia (Zhu et al. 2019; Iqubal et al. 2020). In the present study, severe damage to bone marrow by CTX was observed in the histopathological lesions. However, the toxicity of CTX was reduced in mice treated with SXF, which resulted in an increased number of bone marrow haematopoietic cells, suggesting that SXF had a role in restoring haematopoietic function. In adults, there were still small numbers of HSCs in the spleen, but in adult animals, the spleen may also be involved in haematopoiesis (known as extramedullary haematopoiesis) if there is an increased demand for peripheral blood, producing RBCs, granulocytes, and platelets (Zhong et al. 2009; Zhang et al. 2019). Our results indicated that SXF increased the splenic index, and histopathological tests also showed that SXF increased the number of splenic red lineage cells and promoted the recovery of splenic function after CTX injury.

In recent years, with the popularization of systems biology, network pharmacology has become an important tool for analyzing the performance of TCM (Zhang et al. 2019; Cui et al. 2020; Sinan et al. 2020). According to the absorption, distribution, and metabolism, excretion analysis (ADME) principle, we predicted and found the main active components of SXF to be flavonoids (e.g., isorhamnetin and luteolin), kaempferol, and amino acids (arginine, histidine, and lysine) (Table S1). The cytotoxic effects of CTX include induction of free radical production and increased oxidative stress. CTX disrupts antioxidant systems, decreases superoxide dismutase (SOD), catalase (CAT), and glutathione peroxidase (GSH-PX) activity, and increases malondialdehyde (MDA) activity (Tripathi and Jena 2009; Athira et al. 2020; Niu et al. 2020). When the oxidative-antioxidant system is imbalanced, it results in common diseases and various ageing diseases. Isorhamnetin has been shown to have anti-inflammatory, anticancer, and antioxidant activities (Shi et al. 2018; Kim et al. 2019). Luteolin has antiallergic, anti-inflammatory, and anticancer functions (Deng et al. 2017), and kaempferol can affect cell survival and apoptosis and has antioxidant effects (Seydi et al. 2018). The GO results of SXF action target showed that the redox activity process was also enriched. In conclusion, our results suggested that SXF may restore haematopoiesis by reducing the oxidative damage caused by CTX to the organism's haematopoietic system, thereby improving the body's ability to resist chemotherapy drug-induced aplastic anaemia.

Haematopoiesis is a dynamic developmental process involving the complex regulation of multiple cellular mechanisms in HSCs, including proliferation, self-renewal, differentiation, and apoptosis to produce adequate numbers of blood cells to maintain homeostasis of human physiological functions (Pinho and Frenette 2019; Lee et al. 2020). The KEGG pathway analysis of SXF’s anti-anaemia effect included pathways in cancer and the HIF-1 and p53 signalling pathways, among others. The HIF-1 signalling pathway is primarily designed to maintain a steady-state of oxygen in the body and is involved in the regulation of angiogenesis and inflammation in haematopoiesis and the regulation of the formation of HSCs in the haematopoietic system (Wierenga et al. 2014; Gerri et al. 2018). p53 has a prodifferentiation effect on HSCs. Activation of the p53 gene induces specific expression of p53 protein in vascular endothelial cells, leading to vascular endothelial cell expansion and thus HSCs fail to maintain dormancy, mobilize to the periphery, and are significantly depleted (Si et al. 2018). In the PPI analysis, 12 of the top 15 targets belonged to the mitogen-activated protein kinase (MAPK) signalling pathway. Interestingly, activation of the MAPK pathway promoted myeloid/granulocyte lineage differentiation, and that overactivation of extracellular regulated protein kinase (ERK) (ERK is a key node in the MAPK pathway) leads to functional HSC failure (Hinge et al. 2017; Tadokoro et al. 2018; Barbosa et al. 2019). IL-6 plays a role in blocking erythropoiesis during the pro-erythropoiesis phase and blocking anti-human IL-6 antibodies can improve acute myeloid leukemia-induced anaemia and extend overall survival (Zhang et al. 2020). FOS is associated with Krueppel-like factor (KLF)1 and KLF9 and synergistically regulates erythropoiesis (Ren et al. 2018). Given the treatment principles of TCM, which indicate multiple components against multiple targets (Nie et al. 2019; Huang et al. 2020), SXF may play a role in restoring haematopoiesis or slow anaemia via, but not limited to, the above genes or pathways, although this makes it difficult to elaborate on the mechanism of action of SXF.

In subsequent studies, the active components of SXF will be further determined by mass spectrometry. According to pharmacological theory, monomers will be used for animal or cell experiments to screen and identify the main active components of SXF. Moreover, related pathways will be analyzed based on the screened targets. Also, the relationship between gut microorganisms and chemical components in TCM has been investigated with an increased understanding (Feng et al. 2019; Gong et al. 2020), which can be based on 16 s or macrogenomic and metabolome sequencing for combined multi-omics analysis to investigate the role of gut microbes in the efficacy of SXF.

## Conclusions

In the present study, SXF increased the RBC count in a CTX induced anaemic female Balb/c mouse model and reduced spleen and bone marrow damage, i.e., slowed CTX-induced anaemia. Network pharmacology results suggested that SXF may restore haematopoietic function by reducing oxidative damage to the body's haematopoietic system caused by CTX. The results showed that SXF was effective in the treatment of aplastic anaemia caused by cyclophosphamide, thus improving the body's ability to resist aplastic anaemia. In conclusion, our results indicated that SXF had some efficacy in the treatment of anaemia. These results provide a reference for further research on the efficacy, mechanism, and possible clinical application of SXF.

## Supplementary Material

Figure_S1.tif

Table_S2.xlsx

Table_S1.xlsx
