# Supplementary figures and images for: Therapeutic effects of Sheng Xue Fang in a cyclophosphamide-induced anaemia mouse model
Source: Pharm Biol. 2021 Jun 27;59(1):787–96. doi: 10.1080/13880209.2021.1941133 (PMC8238071; doi:10.1080/13880209.2021.1941133)

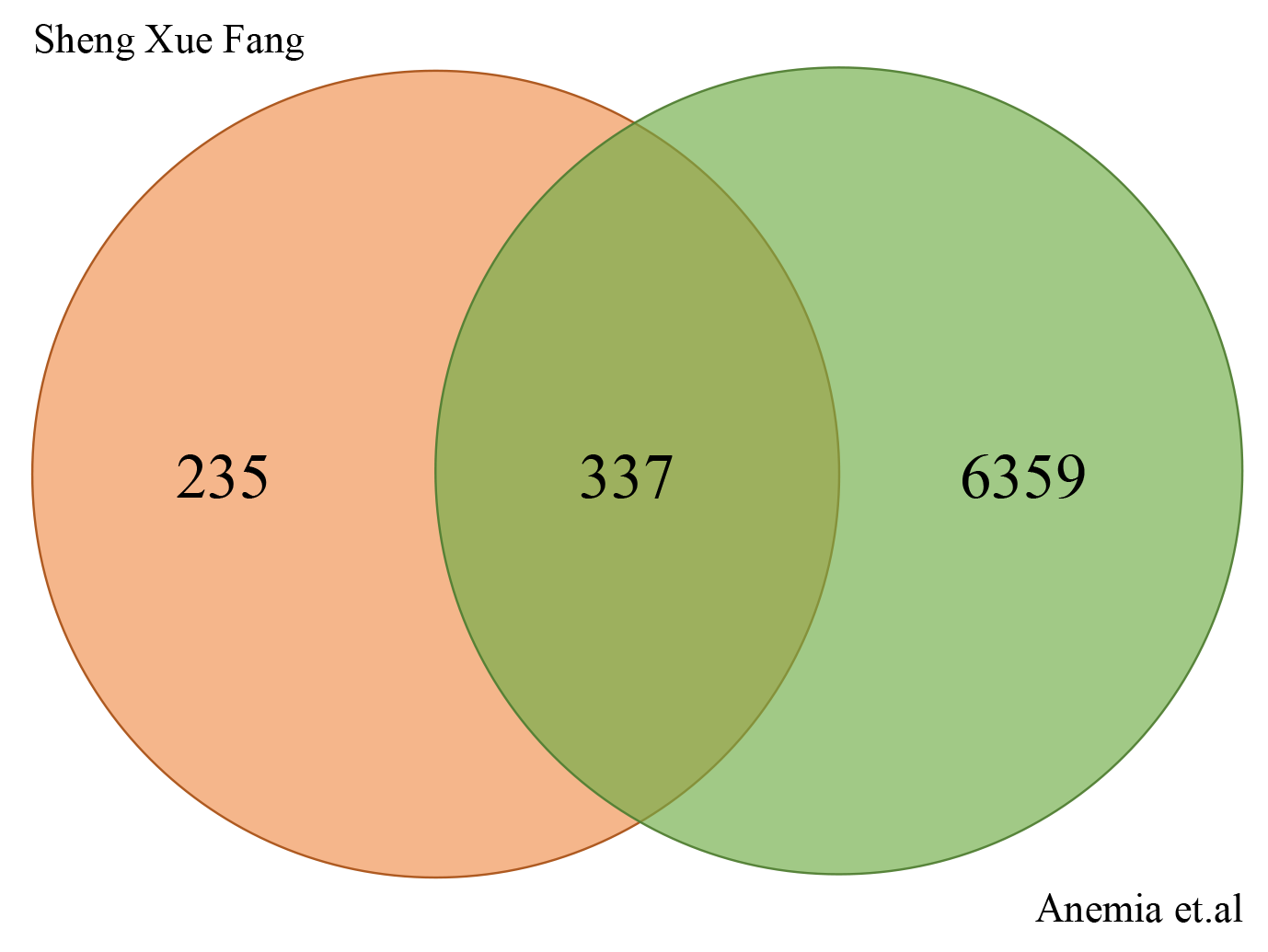

Supplement: Figure_S1.tif [file IPHB_A_1941133_SM8031.tif]
